# Reactive Agility Performance in Older Adults: Associations With Muscle Health and Functional Performance

**DOI:** 10.1111/ggi.70871

**Published:** 2026-10-14

**Authors:** Min Hsu, Fan‐Chen Liu, Chia‐Chi Yang, Yu‐Lin You, Kuei‐Pin Kuo, Lan‐Yuen Guo

**Affiliations:** ^1^ Department of Sports Medicine, College of Medicine Kaohsiung Medical University Kaohsiung Taiwan; ^2^ The Master Program of Long‐Term Care in Aging, College of Nursing Kaohsiung Medical University Kaohsiung Taiwan; ^3^ Department of Sports Medicine China Medical University Taichung Taiwan; ^4^ Office of Physical Education National Ping‐Tung University of Science and Technology Pingtung Taiwan; ^5^ Ph. D. Program in Biomedical Engineering, College of Medicine Kaohsiung Medical University Kaohsiung Taiwan; ^6^ Precision Sports Medicine and Health Promotion Center Kaohsiung Medical University Kaohsiung Taiwan; ^7^ Research Center for Precision Environmental Medicine Kaohsiung Medical University Kaohsiung Taiwan; ^8^ College of Health Sciences Kaohsiung Medical University Kaohsiung Taiwan; ^9^ Center for Long‐Term Care Research Kaohsiung Medical University Kaohsiung Taiwan; ^10^ Department of Medical Research Kaohsiung Medical University Hospital Kaohsiung Taiwan; ^11^ College of Humanities & Social Sciences National Pingtung University of Science and Technology Pingtung Taiwan

**Keywords:** aged, functional performance, muscle strength, reaction time, skeletal muscle

## Abstract

**Introduction:**

This study investigated reactive agility performance in older adults and examined its associations with muscle‐related and functional factors.

**Methods:**

This cross‐sectional study included 114 community‐dwelling older adults with 110 valid reactive‐agility data. A modified functional reactive agility test (FRAT) provided total time (TT), decision time (DT), and movement time (MT) from each participant's shortest‐TT trial. Muscle health was characterized using skeletal muscle index (SMI) and handgrip strength, while functional performance was assessed separately using the five‐times sit‐to‐stand test (5STST). Between‐group differences were tested using one‐way or Welch analysis of variance as appropriate. Separate multivariable models were fitted for TT, DT, and MT, with multiple‐testing correction.

**Results:**

Minimum‐TT trial outcomes did not differ among the four muscle‐health groups. In the multivariable models, slower 5STST performance was consistently associated with longer TT (*β* = 0.48), DT (*β* = 0.37), and MT (*β* = 0.44), with all associations remaining significant after false‐discovery‐rate correction (all *q* < 0.001). No other examined covariate remained significantly associated with reactive agility outcomes after multiple‐testing correction. Variance decomposition of TT showed that the MT variance term constituted the largest component of between‐participant variability (70.2%), compared with the DT variance (10.4%) and DT–MT covariance (19.4%) terms.

**Conclusion:**

Reactive agility did not differ significantly among muscle‐health groups, whereas 5STST performance was consistently associated with TT, DT, and MT. Movement time represented the largest component of between‐participant variability in TT, suggesting that functional performance and temporal components may offer complementary information for characterizing reactive agility in older adults.

## Graphical abstract

In community‐dwelling older adults, longer five‐times sit‐to‐stand test time was independently associated with longer total, decision, and movement times during the modified functional reactive agility test. Movement‐time variance represented the largest component of between‐participant total‐time variance in this sample, whereas reactive‐agility outcomes did not differ significantly among muscle‐health groups.

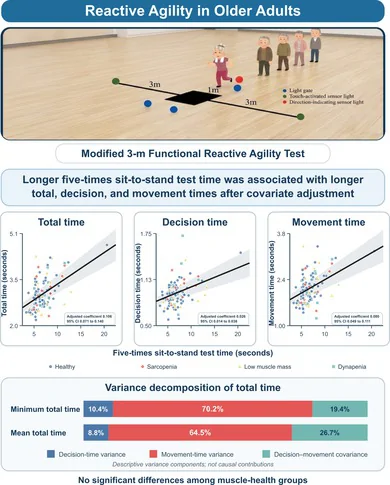

## 1 Introduction

As populations age worldwide, falls represent an increasingly important public health concern because older adults experience a substantial burden of fall‐related injury, disability, and mortality [1]. Approximately one‐third of individuals aged 65 years and older experience at least one fall annually, with the incidence increasing to 40% among those aged 75 years and older and up to 50% among those aged 80 years and older [2]. Age‐related declines in muscle mass, muscle strength, and movement control contribute substantially to impaired mobility and increased fall risk in older adults. Previous studies have shown that muscle mass and strength progressively decline with aging and are associated with adverse outcomes such as disability, hospitalization, and mortality [3, 4, 5]. These findings highlight the importance of identifying functional impairments related not only to muscle loss itself but also to the ability to effectively control and adapt movement during daily activities.

Muscle health in older adults is increasingly recognized as an important determinant of physical function and mobility. Conditions such as sarcopenia and dynapenia are commonly used to characterize different muscle health phenotypes based on established criteria such as those proposed by the Asian Working Group for Sarcopenia (AWGS) [6]. In particular, dynapenia, characterized by reduced muscle strength despite relatively preserved muscle mass, has been consistently associated with impaired functional performance, disability, and increased fall risk [7]. Although muscle health classifications provide important information regarding functional decline and prognosis, most assessments and interventions for older adults continue to focus primarily on muscle quantity, strength, or pre‐planned physical performance tasks, with relatively limited attention given to agility‐related performance, particularly reactive agility [8, 9].

Reactive agility, defined as the ability to respond quickly to external stimuli by integrating perception, decision‐making, and movement execution [10]. Unlike conventional clinical assessments such as the Timed Up and Go test, 10‐step test, and four‐square step test, which primarily evaluate pre‐planned and predictable movements, reactive agility tasks require individuals to adapt their movements in response to unforeseen environmental challenges [11, 12, 13, 14]. Such abilities may be particularly relevant for older adults, as real‐world mobility and fall avoidance often depend on rapid lower‐limb responses and adaptive movement control, and timely decision‐making [15]. Recent studies have demonstrated that functional reactive agility tests (FRAT) possess acceptable reliability and construct validity [16, 17]. Furthermore, reactive agility performance is considered a multidimensional ability influenced by both physical and cognitive factors, including muscle strength, lower‐extremity functional performance, reaction time, perceptual processing, and decision‐making, with previous studies suggesting that decision‐related processes may account for a substantial proportion of performance variability [18, 19]. These findings indicate that different components of reactive agility, such as decision‐related and movement‐related processes, may be influenced by different physiological and functional factors.

However, research examining reactive agility performance in older adults remains limited, particularly regarding muscle health status and functional performance. Furthermore, while FRAT has been proposed and validated in younger populations, its application in older adults remains limited. More importantly, few studies have separately investigated decision‐related and movement‐related components of reactive agility in older adults. Therefore, this study aimed to investigate reactive agility performance using a FRAT in community‐dwelling older adults and to determine the independent contributions of muscle mass, muscle strength, and functional performance to reactive agility components, including decision time, movement time, and total performance time.

## 2 Materials and Methods

### 2.1 Study Design

This cross‐sectional observational study investigated reactive agility performance in community‐dwelling older adults and its associations with muscle health and functional capacity. Participants were recruited from community centers and local community settings. All assessments were performed during a single session and included measurements of body composition, muscle strength, functional performance, perceived concern about falling, and reactive agility. The order of assessments was standardized for all participants, and sufficient rest periods were provided between tests to minimize fatigue.

### 2.2 Participants and Sample Size

Participants were recruited from community centers and local community settings between 2025/9 and 2025/12. Inclusion criteria were as follows: community‐dwelling older adults aged 65–80 years who were able to communicate verbally or in writing and capable of completing the study procedures. Exclusion criteria included participants being unable to understand and follow verbal or written instructions, chest pain during recent physical activity, angina or joint pain limiting movement, congestive heart failure, medical advice against exercise participation, ongoing cancer treatment, neuromuscular disorders, musculoskeletal conditions affecting the lower extremities, and physician‐diagnosed severe osteoporosis. This study was conducted in accordance with the Declaration of Helsinki and approved by the Institutional Review Board of Kaohsiung Medical University Hospital (KMUHIRB‐E(I)‐20250028). This study was registered at ClinicalTrials.gov (Identifier: NCT07375888). The participants signed an informed consent form prior to their participation in the study.

A priori sample size estimation was performed using G*Power (version 3.1), assuming a medium effect size (*f* = 0.25), an alpha level of 0.05, and a statistical power of 0.80 for comparisons across four groups. The estimated required sample size was 176 participants. However, due to practical constraints in participant recruitment, the final sample size was smaller than the target. Recruitment resulted in 114 participants, with 110 providing valid reactive‐agility data. The limited sample size and unequal group sizes were considered by presenting effect estimates with 95% confidence intervals and interpreting the between‐group differences based on their precision.

### 2.3 Procedure

All participants completed the assessments in a single session under the supervision of two trained researchers. Upon arrival, participants first completed informed consent procedures and a basic demographic questionnaire. Subsequently, physiological measurements and physical performance tests were conducted, followed by the assessment of perceived concern about falling and reactive agility. Participants were divided into four groups—healthy, dynapenia, low muscle mass, and sarcopenia—based on muscle mass and strength parameters following the Asian Working Group for Sarcopenia (AWGS) 2019 criteria. The order of assessments was standardized for all participants, and adequate rest periods were provided between tests to minimize fatigue.

### 2.4 Measurement

#### 2.4.1 Body Composition

Body composition was assessed using a multi‐frequency bioelectrical impedance analyzer (BIA) (InBody 970; InBody Co. Ltd., Seoul, South Korea). Participants were instructed to refrain from drinking water for at least 1 h before measurement. They stood barefoot on the device and held the hand electrodes during measurement. Appendicular skeletal muscle mass was measured using BIA assessment, and the skeletal muscle index (SMI) was computed as the appendicular skeletal muscle mass divided by the height squared (kg/m^2^). Additionally, height, body weight, and body mass index (BMI) were documented.

#### 2.4.2 Handgrip Strength

Handgrip strength was measured using a handheld dynamometer (Jamar hydraulic hand dynamometer; Patterson Medical, Warrenville, IL, USA). Participants were seated with the elbow flexed at 90° without arm support [20]. Three trials were performed, and the maximum value was used for analysis.

#### 2.4.3 Five‐Times Sit‐To‐Stand Test

Lower‐limb functional performance was evaluated through the five‐times sit‐to‐stand test (5STST). Participants sat on a standard chair with arms crossed over their chests and were asked to stand up and sit down five times as fast as possible without using their hands [21]. The total time to complete the task was recorded.

#### 2.4.4 Falls Efficacy Scale International

Concern about falling was assessed using the Taiwan Chinese version of Falls Efficacy Scale International (FES‐I) [22]. This 16‐item questionnaire evaluates concern about falling during daily activities, with each item scored on a 4‐point scale. Total scores range from 16 to 64, with higher scores indicating greater fear of falling.

#### 2.4.5 Reactive Agility Test

Reactive agility was assessed using a modified functional reactive agility test (FRAT) [16]. A visual stimulus and timing system (Sportreact, Zagreb, Croatia), with 1‐millisecond resolution, was used to deliver cues and record response times. Before testing, participants completed a standardized 3‐min warm‐up and two familiarization trials. Participants were instructed to stand upright and barefoot in the designated starting zone, facing the visual stimulus device ahead before the cue was presented. Two initial timing gates were positioned at the left and right boundaries of the starting area, 1 m apart, and target sensors were positioned 3 m to the left and right (Figure 1).

**FIGURE 1:**
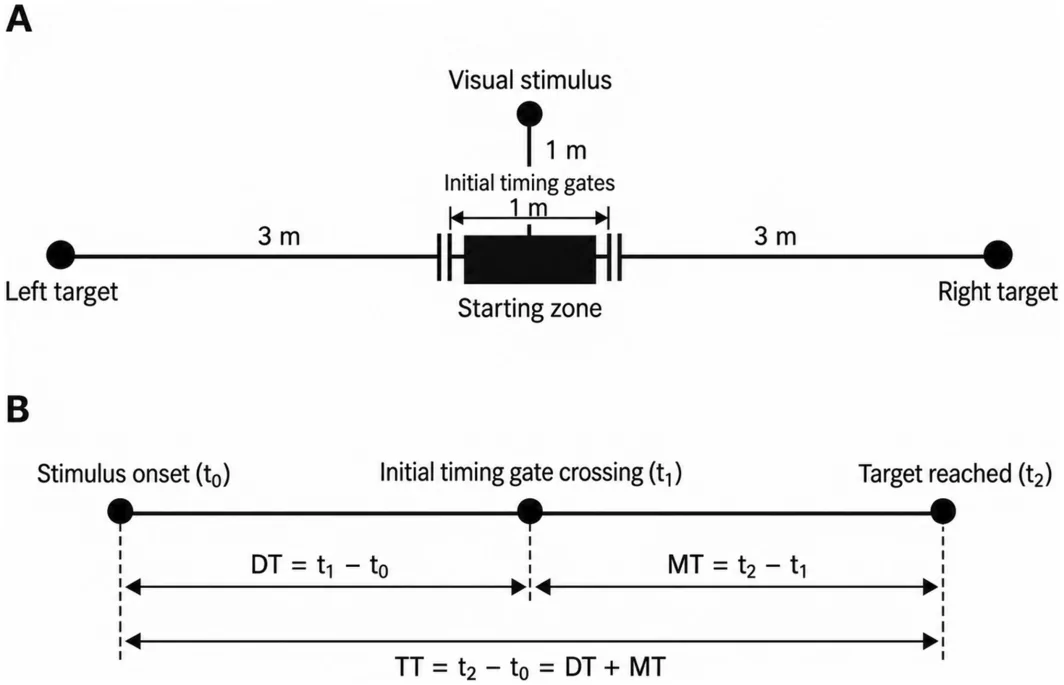
Modified functional reactive agility test. (A) Layout: Visual stimulus 1 m ahead, lateral timing gates 1 m apart, and laser‐equipped targets 3 m to either side. (B) Timing: T0, stimulus onset; t1, timing‐gate crossing; t2, target‐sensor activation. Decision time (DT) = t1 − t0; movement time (MT) = t2 − t1; total time (TT) = t2 − t0 = DT + MT.

At the start of each trial, the system randomly displayed a left or right visual cue with no predetermined number of trials in either direction, and participants were instructed to move as quickly as possible toward and contact the indicated target. Each target was equipped with a laser sensor that detected the participant's arrival and recorded the target‐reaching time (t2). Decision time (DT) was the interval from stimulus onset (t0) to crossing the corresponding initial timing gate (t1). Movement time (MT) was the interval from timing‐gate crossing (t1) to laser‐sensor detection at the target (t2). Total time (TT) was calculated within each trial as TT = DT + MT. Each participant completed 10 recorded trials. Trials were deemed invalid if the participant moved incorrectly or did not activate the target sensor properly. These invalid trials were excluded from the analysis and were not repeated. There was no set rest period between trials.

The present protocol differed from the original FRAT in two respects. First, the movement distance was extended from 1.5 to 3 m. Second, the outcome‐selection procedure was modified. Whereas the original FRAT used the fastest DT, MT, and TT values obtained across separate trials [16], this study selected the trial with the shortest TT and used the DT and MT from that same trial, ensuring all three metrics came from a single performance. Therefore, this procedure is a modified version of the FRAT. While within‐sample reliability was assessed, the measurement properties valid for the original FRAT cannot be assumed to generalize to the modified 3‐m protocol. Additionally, its construct and criterion validity in older adults remain unestablished.

### 2.5 Muscle Health Classification

Participants were classified into four study‐defined muscle‐health phenotypes using BIA‐derived SMI and handgrip strength thresholds from the Asian Working Group for Sarcopenia (AWGS) 2019 criteria [6]. Low muscle mass was defined as a skeletal muscle index (SMI) < 7.0 kg/m^2^ in men and < 5.7 kg/m^2^ in women. Low muscle strength was defined as handgrip strength < 28 kg in men and < 18 kg in women. Based on these criteria, participants were categorized into four groups: (1) healthy (normal muscle mass and strength), (2) dynapenia (low muscle strength with normal muscle mass), (3) low muscle mass (low muscle mass with normal muscle strength), and (4) sarcopenia (low muscle mass and low muscle strength).

### 2.6 Statistical Analysis

All statistical analyses were performed using SPSS software (version 30.0; SPSS Inc., Chicago, USA), with the significance level set at *α* = 0.05. Continuous variables are presented as mean ± standard deviation. Normality was assessed using the Shapiro–Wilk test, and homogeneity of variance was assessed using Levene's test. Trial‐to‐trial reliability was evaluated using two‐way random‐effects, absolute‐agreement intraclass correlation coefficients for a single trial [ICC(A,1)] and the mean of 10 trials [ICC(A,10)], with 95% bootstrap confidence intervals. One‐way analysis of variance (ANOVA) was used for the primary between‐group comparisons when homogeneity of variance was supported. Welch ANOVA was specified for unequal variances. Because several variables showed departures from normality and the group sizes were unequal, Kruskal–Wallis tests were additionally performed as nonparametric sensitivity analyses. Effect sizes are reported as eta squared (*η* ^2^) with 95% confidence intervals. Categorical associations were examined using the chi‐square test or the Fisher–Freeman–Halton exact test when expected cell counts were small.

Bivariate associations between agility outcomes and participant characteristics were examined across the entire sample using two‐sided Spearman rank correlation coefficients because of the non‐normal distribution of several variables and the presence of potentially influential observations. These analyses were considered exploratory and were not used for primary statistical inference; therefore, their *p*‐values were not adjusted for multiple comparisons. Between‐participant variability in TT was described using Var(TT) = Var(DT) + Var(MT) + 2Cov(DT, MT). The variance terms represent the variability of DT and MT, whereas the covariance term reflects their joint variation. Each term was expressed as a percentage of TT variance for descriptive purposes.

Separate multiple linear regression models were specified as the primary association analyses for TT, DT, and MT. Separate models were fitted for each outcome, with covariates selected a priori based on their demographic or clinical relevance and entered simultaneously. Regression coefficients, 95% confidence intervals, and model fit statistics were reported, and standard diagnostics were used to assess model assumptions, multicollinearity, and influential observations. To account for multiple testing across the three primary regression models, the Benjamini–Hochberg procedure was applied to all 18 regression coefficient tests, with an FDR‐adjusted *q* value < 0.05 considered statistically significant. Sensitivity analyses, including between‐group comparisons and regression models, were repeated using mean trial values instead of the primary shortest‐TT trial outcomes. The stability of the regression estimates was further evaluated using 5000‐sample bias‐corrected and accelerated bootstrap confidence intervals and by refitting each model after excluding the observation with the highest Cook's distance.

## 3 Result

### 3.1 Participant Characteristics

A total of 114 older adults (31 males and 83 females) were included in the study. Based on BIA‐derived SMI and handgrip strength, 69 participants were classified as healthy, 12 as having dynapenia, 22 as having low muscle mass, and 11 as having sarcopenia. SMI and handgrip strength, which were used to define muscle‐health status according to sex‐specific criteria, are presented descriptively in Table 1. BMI differed significantly among the four muscle‐health groups (*p* < 0.001). Tukey HSD post hoc comparisons showed that BMI was significantly lower in the sarcopenia group than in the healthy (*p* = 0.03) and dynapenia groups (*p* = 0.02), and lower in the low muscle mass group than in the healthy (*p* = 0.02) and dynapenia groups (*p* = 0.02). No significant differences were observed between the healthy and dynapenia groups or between the sarcopenia and low muscle mass groups. No statistically significant association was observed between sex and muscle‐health status (Table 1).

**TABLE 1:** Participant characteristics according to muscle health status.

Variables	Group						
	Healthy group (*n* = 69)	Dynapenia group (*n* = 12)	Low muscle mass group (*n* = 22)	Sarcopenia group (*n* = 11)	*F*	*p*	Effect size (95% CI)
	Mean ± SD	Mean ± SD	Mean ± SD	Mean ± SD			
Age, years	71.33 ± 3.43	72.42 ± 3.50	72.05 ± 3.79	71.64 ± 3.14	0.48	0.70^a^	*η* ^2^ = 0.01 (0.00–0.05)
Male sex, *n* (%)	16 (23.2)	4 (33.3)	7 (31.8)	4 (36.4)	—	0.62^c^	V = 0.11
Height (m)	1.60 ± 0.07	1.58 ± 0.07	1.57 ± 0.07	1.56 ± 0.09	1.58	0.20^a^	*η* ^2^ = 0.04 (0.00–0.13)
Weight (kg)	59.63 ± 9.12	61.63 ± 9.88	52.07 ± 7.30	49.95 ± 8.33	7.76	< 0.001^a^	*η* ^2^ = 0.17 (0.05–0.28)
BMI, kg/m^2^	23.41 ± 3.19	24.46 ± 4.08	21.18 ± 2.58	20.53 ± 2.70	5.83	< 0.001^a^	*η* ^2^ = 0.14 (0.03–0.24)
Muscle mass (kg)	16.89 ± 3.13	17.00 ± 3.38	14.30 ± 2.72	14.12 ± 3.30	5.79	0.001^a^	*η* ^2^ = 0.14 (0.03–0.24)
SMI, kg/m^2^	6.40 ± 0.88	6.78 ± 0.98	6.01 ± 0.58	6.26 ± 0.75	2.42	0.07^a^	*η* ^2^ = 0.06 (0.00–0.15)
Maximum handgrip strength, kg	25.10 ± 6.69	18.00 ± 5.41	23.64 ± 6.01	18.09 ± 5.15	7.19	< 0.001^a^	*η* ^2^ = 0.16 (0.04–0.27)
5STST time, s	6.60 ± 2.35	7.22 ± 1.76	7.41 ± 2.68	7.47 ± 2.26	1.00	0.40^a^	*η* ^2^ = 0.03 (0.00–0.09)
FES‐I score	25.26 ± 8.01	31.75 ± 13.94	24.41 ± 8.13	25.09 ± 7.60	0.90	0.45^b^	*η* ^2^ = 0.06 (0.00–0.13)
Minimum total time, s	3.02 ± 0.49	3.17 ± 0.50	3.14 ± 0.62	3.22 ± 0.57	0.81	0.49^a^	*η* ^2^ = 0.02 (0.00–0.08)
Decision time from the minimum‐TT trial, s	0.92 ± 0.14	1.03 ± 0.29	0.94 ± 0.16	1.01 ± 0.14	1.79	0.15^a^	*η* ^2^ = 0.05 (0.00–0.13)
Movement time from the minimum‐TT trial, s	2.09 ± 0.40	2.14 ± 0.51	2.20 ± 0.50	2.21 ± 0.47	0.48	0.70^a^	*η* ^2^ = 0.01 (0.00–0.06)

*Note:* Data are presented as mean ± standard deviation or number (percentage). Effect sizes are presented as eta squared (*η* ^2^) with 95% confidence intervals for continuous variables and Cramer's *V* for sex. Group sample sizes for the minimum‐TT trial outcomes were 67 healthy, 11 dynapenia, 21 low muscle mass, and 11 sarcopenia participants.

Abbreviations: 5STST, five‐times sit‐to‐stand test; BMI, body mass index; FES‐I, Falls Efficacy Scale‐International; SMI, skeletal muscle index; TT, total time.

^a^ One‐way ANOVA.

^b^ Welch ANOVA.

^c^ Fisher–Freeman–Halton exact test.

### 3.2 Reactive Agility Performance Across Muscle Health Groups

Valid FRAT outcomes were available for 110 participants, including 67 healthy participants, 11 with dynapenia, 21 with low muscle mass, and 11 with sarcopenia. The final trial from one participant was excluded because of a confirmed recording‐process error; this participant retained nine valid trials and remained in analyses for which valid measurements were available. Levene's tests supported homogeneity of variance for TT, DT, and MT (all *p* > 0.05). No significant between‐group differences were observed in minimum TT, DT, or MT, and the corresponding effect sizes were small (*η* ^2^ = 0.01–0.05; Table 1). Sensitivity analyses using Welch ANOVA and Kruskal–Wallis tests yielded the same conclusions. Analyses based on mean trial values also showed no significant between‐group differences. Detailed results from the Welch ANOVA, Kruskal–Wallis, and mean‐trial sensitivity analyses are provided in Table S1.

Among the 109 participants with 10 valid trials, ICC(A,1) was 0.59 (95% bootstrap CI 0.29–0.81) for TT, 0.30 (0.09–0.46) for DT, and 0.63 (0.40–0.81) for MT. ICC(A,10) was 0.94 (0.80–0.98) for TT, 0.81 (0.51–0.89) for DT, and 0.94 (0.87–0.98) for MT.

### 3.3 Correlation Analysis

Across the full analytic sample (*n* = 110), two‐sided Spearman rank correlations showed that longer 5STST time was associated with longer TT (*r* = 0.45, *p* < 0.001), DT (*r* = 0.36, *p* < 0.001), and MT (*r* = 0.42, *p* < 0.001). Older age was associated with longer TT (*r* = 0.316, *p* < 0.001) and MT (*r* = 0.32, *p* < 0.001), but not with DT (*r* = 0.15, *p* = 0.13). Greater handgrip strength was associated with shorter TT (*r* = −0.24, *p* = 0.01) and DT (*r* = −0.32, *p* < 0.001), whereas its association with MT was not statistically significant (*r* = −0.18, *p* = 0.06). Neither SMI (*r* = 0.05–0.11, *p* = 0.24–0.61) nor FES‐I score (*r* = 0.05–0.08, *p* = 0.41–0.62) was correlated with TT, DT, or MT. These correlations were exploratory, and the reported *p*‐values were not adjusted for multiple comparisons.

For the within‐trial decomposition of TT, the variance in decision time, the variance in movement time, and twice the covariance between decision time and movement time represented 10.4%, 70.2%, and 19.4% of the total variance, respectively. The corresponding proportions for the mean trial values were 8.8%, 64.5%, and 26.7% (Figure 2).

**FIGURE 2:**
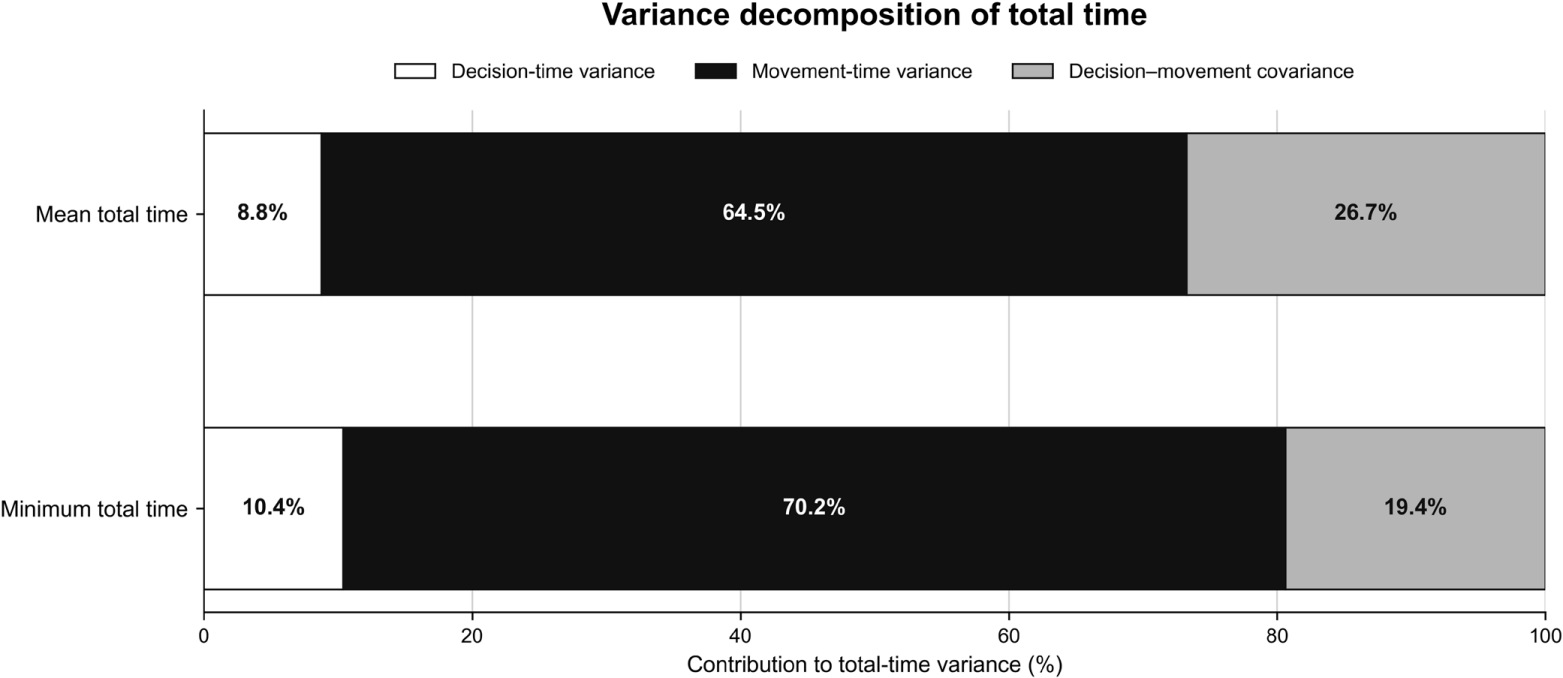
Variance decomposition of minimum and mean total time. Stacked bars show decision‐time variance, movement‐time variance, and twice their covariance as percentages of between‐participant total‐time variance (summing to 100%).

### 3.4 Multiple Regression Analysis

The regression model for total time accounted for 37.3% of the variance (adjusted *R* ^2^ = 0.34; *F*(6,103) = 10.22, *p* < 0.001). The corresponding values were 27.9% for decision time (adjusted *R* ^2^ = 0.24; *F*(6,103) = 6.64, *p* < 0.001) and 29.0% for movement time (adjusted *R* ^2^ = 0.25; *F*(6,103) = 7.01, *p* < 0.001) (Table S2). After simultaneous adjustment for age, sex, handgrip strength, skeletal muscle index, and FES‐I score, each 1 s longer 5STST time was associated with a 0.11 s longer total time (95% CI 0.07–0.14), a 0.03 s longer decision time (95% CI 0.01–0.04), and a 0.08 s longer movement time (95% CI 0.05–0.11). All three associations remained statistically significant after FDR correction (all *p* < 0.001; all *q* < 0.001; Figure 3). Before multiple‐testing correction, age was associated with total time (*B* = 0.03, 95% CI 0.005–0.06; *p* = 0.02) and decision time (*B* = 0.01, 95% CI 0.00–0.02; *p* = 0.04). The coefficients for the remaining covariates are shown in Table 2.

**FIGURE 3:**
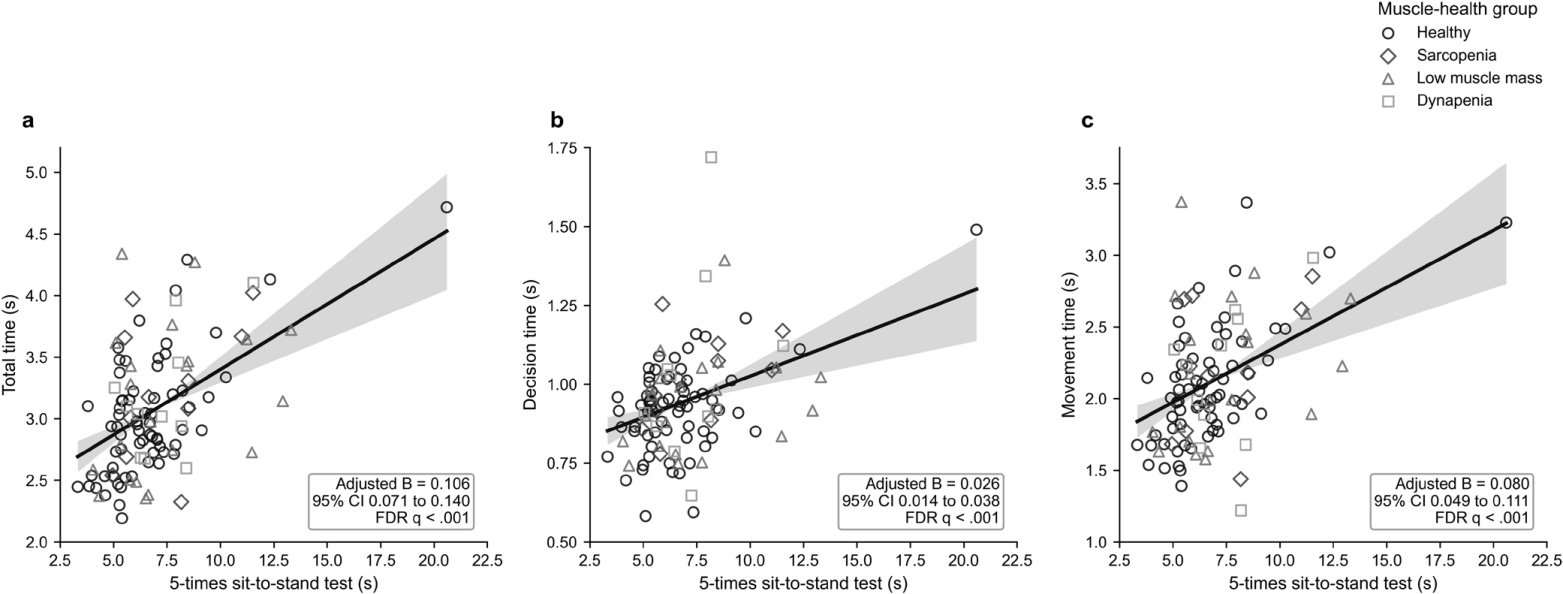
Associations of 5STST time with (a) total, (b) decision, and (c) movement time from the minimum‐total‐time trial in the modified FRAT. Symbols represent participants based on muscle‐health status, while lines and bands depict unadjusted fits and 95% CIs. Insets provide adjusted coefficients, 95% CIs, and FDR‐corrected *q* values.

**TABLE 2:** Primary multiple linear regression models for same‐trial reactive‐agility outcomes.

Outcome (*N* = 110)	Predictor	*B* (SE)	*β*	95% CI for B	*p*	FDR‐adjusted *q*
Total time	Age	0.031 (0.013)	0.204	0.005 ~ 0.056	0.018	0.081
	Sex	−0.082 (0.136)	−0.070	−0.352 ~ 0.188	0.549	0.706
	5STST	0.106 (0.017)	0.484	0.071 ~ 0.140	< 0.001	< 0.001
	Handgrip strength	−0.016 (0.009)	−0.209	−0.033 ~ 0.001	0.071	0.183
	SMI	0.042 (0.049)	0.069	−0.054 ~ 0.138	0.388	0.617
	FES‐I	−0.002 (0.005)	−0.035	−0.011 ~ 0.007	0.658	0.715
Decision time	Age	0.009 (0.004)	0.187	0.000 ~ 0.018	0.042	0.151
	Sex	0.018 (0.047)	0.049	−0.075 ~ 0.112	0.699	0.715
	5STST	0.026 (0.006)	0.366	0.014 ~ 0.038	< 0.001	< 0.001
	Handgrip strength	−0.005 (0.003)	−0.208	−0.011 ~ 0.001	0.094	0.212
	SMI	0.006 (0.017)	0.031	−0.027 ~ 0.039	0.715	0.715
	FES‐I	0.001 (0.002)	0.037	−0.002 ~ 0.004	0.667	0.715
Movement time	Age	0.022 (0.011)	0.171	−0.001 ~ 0.044	0.060	0.180
	Sex	−0.100 (0.122)	−0.103	−0.341 ~ 0.141	0.411	0.617
	5STST	0.080 (0.016)	0.436	0.049 ~ 0.111	< 0.001	< 0.001
	Handgrip strength	−0.011 (0.008)	−0.169	−0.026 ~ 0.005	0.168	0.336
	SMI	0.036 (0.043)	0.070	−0.050 ~ 0.122	0.408	0.617
	FES‐I	−0.003 (0.004)	−0.056	−0.011 ~ 0.005	0.508	0.703

*Note:* Values are unstandardized regression coefficients (*B*) with standard errors (SE), standardized coefficients (*β*), 95% confidence intervals (CI), unadjusted *p*‐values, and Benjamini–Hochberg FDR‐adjusted *q*‐values. Sex was coded as 1 for male and 2 for female; the sex coefficient therefore represents the adjusted difference for women relative to men. The Benjamini–Hochberg procedure was applied across the 18 coefficient tests; *q* < 0.05 indicated statistical significance.

Abbreviations: 5STST, five‐times sit‐to‐stand test; DT, decision time; FES‐I, Falls Efficacy Scale‐International; MT, movement time; SMI, skeletal muscle index; TT, total time.

VIF values ranged from 1.04 to 2.24. Residual plots showed no substantial deviations from linearity or homoscedasticity, although departures from normality were observed in the distribution tails. The positive associations between 5STST time and all three agility outcomes persisted when we analyzed mean trial values and after excluding the participant with the largest Cook's distance in all three models. Bootstrap 95% bias‐corrected and accelerated confidence intervals for the three 5STST coefficients excluded zero (Tables S2–S4).

## 4 Discussion

The present study examined reactive agility performance in community‐dwelling older adults with different muscle‐health phenotypes using a modified FRAT. Three main findings emerged. First, no statistically significant differences in reactive agility performance with TT, DT, or MT were observed among the four muscle‐health groups. Second, 5STST performance was consistently associated with TT, DT, and MT in the multivariable models and remained significant after false‐discovery‐rate correction. In contrast, SMI, handgrip strength, sex, and concern about falling assessment were not independently associated with reactive agility outcomes after adjustment for multiple testing. Third, variance decomposition showed that the MT variance term constituted the largest component of between‐participant variability in TT, with a similar pattern observed when mean trial values were analyzed. Collectively, these findings suggest that reactive agility performance in this sample was more consistently associated with lower‐extremity functional performance than with the individual muscle‐related measures examined in the present study.

The consistent association between 5STST performance and all three reactive agility outcomes is a central finding of the present study. The 5STST is an integrated functional task requiring coordinated lower‐extremity force generation, postural control, and movement execution, capacities that may also be relevant to the rapid whole‐body responses required during reactive agility [18]. Previous research in older adults has linked agility performance with lower‐extremity power and other measures of functional capacity. For example, Manderoos et al. reported that lower‐extremity power and age were important determinants of agility performance in older men [23]. Consistent with this concept, the previous study has demonstrated that impaired lower‐extremity function is associated with slower reactive stepping and reduced fall‐avoidance ability in older adults [24]. This interpretation is also consistent with evidence that sit‐to‐stand performance reflects multiple domains of lower‐extremity functional capacity [25, 26]. However, the association with DT should not be interpreted as specifically reflecting cognitive performance, as DT also includes movement before timing‐gate crossing.

In contrast, reactive agility did not differ significantly among the four muscle‐health groups, and neither SMI nor handgrip strength showed independent associations with TT, DT, or MT after adjustment for multiple testing. These findings do not imply that muscle characteristics are unrelated to agility performance. Rather, the muscle‐health classifications used in the present study were based on muscle mass and handgrip strength, which may not fully represent the lower‐extremity functional capacities required for rapid whole‐body movement [27]. This distinction may also help reconcile our findings with those of Manderoos et al. who identified lower‐extremity power, rather than muscle mass or handgrip strength, as an important determinant of agility performance in older men [23]. Together, these findings suggest that integrated lower‐extremity functional performance may be more closely associated with reactive agility than the muscle‐health indicators examined in the present study.

The temporal composition of reactive agility provides additional insight into performance in older adults. In the present study, the MT variance term constituted the largest component of between‐participant variability in TT, with the same pattern observed using mean trial values. Young and Willey reported a different pattern in young, semi‐professional Australian football players performing a reactive agility test with substantial locomotor demands [18, 28]. While these findings were derived using different analytical approaches, the contrasting patterns suggest that the temporal characteristics of reactive agility may vary across populations and task configurations. The greater movement‐related variability observed in the present sample may reflect both the functional characteristics of older adults and the demands of the modified 3‐m protocol, as movement and total times were longer than those reported with the original 1.5‐m FRAT [16]. Taken together, these findings highlight the value of examining the temporal components of reactive agility rather than interpreting overall performance time alone, particularly when comparing populations with different functional characteristics.

### 4.1 Study Limitation

Several limitations should be considered when interpreting these findings. First, the cross‐sectional design precludes causal inference regarding the associations between functional performance and reactive agility. Second, the relatively small and unequal muscle‐health groups, particularly the dynapenia and sarcopenia groups, may have limited the precision of between‐group comparisons; therefore, the absence of statistically significant group differences should not be interpreted as evidence of equivalence. Third, the FRAT was modified by extending the movement distance from 1.5 to 3 m and by using a same‐trial outcome‐selection procedure. Although within‐sample reliability was evaluated, the measurement properties established for the original FRAT cannot be assumed to generalize to this modified protocol, and its construct and criterion validity remain to be established. Finally, DT represents the interval from stimulus onset to timing‐gate crossing and therefore incorporates both perceptual–decision processes and movement before gate crossing; it should not be interpreted as an isolated measure of cognitive decision‐making. In addition, the estimated temporal composition of TT may depend on how repeated trials are summarized. Although analyses based on mean trial values produced the same qualitative pattern as the primary shortest‐TT approach, the relative variance components differed numerically between the two methods. Future studies should validate the modified FRAT in larger and more diverse samples and incorporate direct measures of cognitive, sensory, and neuromuscular function to better characterize reactive agility in older adults.

## 5 Conclusion

In conclusion, reactive agility performance did not differ significantly among muscle‐health groups in this sample of community‐dwelling older adults, whereas 5STST performance was consistently associated with TT, DT, and MT after adjustment for multiple testing. Movement time also represented the largest component of between‐participant variability in total time. These findings suggest that integrated lower‐extremity functional performance may be more closely related to reactive agility than the muscle mass and handgrip strength measures examined here, while temporal‐component analysis may provide information beyond total performance time alone. Further studies using larger samples and validated reactive agility protocols are needed to confirm these findings.

## Author Contributions

Conceptualization: Min Hsu, Fan‐Chen Liu, Chia‐Chi Yang, Yu‐Lin You, Kuei‐Pin Kuo, and Lan‐Yuen Guo. Methodology: Min Hsu, Fan‐Chen Liu, Chia‐Chi Yang, Kuei‐Pin Kuo, and Lan‐Yuen Guo. Formal analysis: Min Hsu, Fan‐Chen Liu, Yu‐Lin You, Kuei‐Pin Kuo, and Lan‐Yuen Guo. Investigation: Min Hsu, Fan‐Chen Liu, Kuei‐Pin Kuo, and Lan‐Yuen Guo. Writing – original draft: Min Hsu, Fan‐Chen Liu, Chia‐Chi Yang, Kuei‐Pin Kuo, and Lan‐Yuen Guo. Writing – review and editing: Min Hsu, Yu‐Lin You, Kuei‐Pin Kuo, and Lan‐Yuen Guo. Funding acquisition: Kuei‐Pin Kuo and Lan‐Yuen Guo.

## Funding

This study was supported by the Kaohsiung Medical University–National Pingtung University of Science and Technology Joint Research Project (grant number NPUSTKMU‐114‐P006), the Ministry of Science and Technology, Taiwan (NSTC114‐2410‐H‐037‐011‐MY2), the Precision Sports Medicine and Health Promotion Center, Kaohsiung Medical University, Kaohsiung 807, Taiwan (KMU‐TC115A06‐1), the Research Center for Precision Environmental Medicine, Kaohsiung Medical University, Kaohsiung, Taiwan from The Featured Areas Research Center Program within the framework of the Higher Education Sprout Project by the Ministry of Education (MOE) in Taiwan, and Kaohsiung Medical University Research Center Grant (KMU‐TC115A01).

## Ethics Statement

All the study procedures were conducted in accordance with the principles outlined in the Declaration of Helsinki. Ethical approval for this study was obtained from the Institutional Review Board of Kaohsiung Medical University Hospital (KMUHIRB‐E(I)‐20 250 028). This study was registered at ClinicalTrials.gov (Identifier: NCT07375888). The participants signed an informed consent form prior to their participation in the study.

## Consent

The authors have nothing to report.

## Conflicts of Interest

The authors declare no conflicts of interest.

## Supporting information


**Table S1:** Sensitivity analyses of between‐group differences in reactive agility.
**Table S2:** Multiple linear regression models using mean trial values.
**Table S3:** Bootstrap estimates for the primary regression models.
**Table S4:** Primary regression models after exclusion of one participant.

## Data Availability

Data supporting this study are available from the corresponding author upon reasonable request.
