# Supplementary material for: Reactive Agility Performance in Older Adults: Associations With Muscle Health and Functional Performance
Source: Geriatr Gerontol Int. 2026 Oct 14;26(10):e70871. doi: 10.1111/ggi.70871 (PMC13668733; doi:10.1111/ggi.70871)
Supplement: Supplementary file 1 [file GGI-26-0-s001.docx]

Supplementary Table S1 Sensitivity analyses of between-group differences in reactive agility

Group comparisons were examined using alternative tests and mean values across valid trials.

## A Mean trial values by muscle-health group

| **Outcome, s** | **Healthy (n = 67)** | **Dynapenia (n = 11)** | **Low muscle mass (n = 21)** | **Sarcopenia (n = 11)** |
| --- | --- | --- | --- | --- |
| Total time | 3.247 ± 0.545 | 3.524 ± 0.642 | 3.377 ± 0.700 | 3.489 ± 0.625 |
| Decision time | 1.042 ± 0.162 | 1.072 ± 0.138 | 1.085 ± 0.237 | 1.118 ± 0.164 |
| Movement time | 2.205 ± 0.433 | 2.451 ± 0.540 | 2.293 ± 0.568 | 2.371 ± 0.488 |

## B Variance assessment and alternative between-group tests

| **Scoring and outcome** | **Levene p** | **Welch F (df1, df2)** | **Welch p** | **Kruskal–Wallis H** | **Kruskal–Wallis p** |
| --- | --- | --- | --- | --- | --- |
| Minimum total time | 0.223 | 0.736 (3, 24.606) | 0.540 | 2.243 | 0.524 |
| Corresponding decision time | 0.052 | 1.372 (3, 24.012) | 0.275 | 3.689 | 0.297 |
| Corresponding movement time | 0.250 | 0.413 (3, 24.048) | 0.745 | 1.409 | 0.703 |
| Mean total time | 0.234 | 1.012 (3, 24.213) | 0.405 | 3.327 | 0.344 |
| Mean decision time | 0.792 | 0.784 (3, 25.539) | 0.514 | 2.082 | 0.556 |
| Mean movement time | 0.214 | 0.963 (3, 24.097) | 0.426 | 2.859 | 0.414 |

## C One-way ANOVA for mean trial values

| **Outcome** | **F (df1, df2)** | **p** | **η² (95% CI)** |
| --- | --- | --- | --- |
| Total time | 1.125 (3, 106) | 0.342 | 0.031 (0.000 to 0.095) |
| Decision time | 0.770 (3, 106) | 0.514 | 0.021 (0.000 to 0.076) |
| Movement time | 1.117 (3, 106) | 0.346 | 0.031 (0.000 to 0.095) |

Values in panel A are mean ± SD. All comparisons include 110 participants. Mean values used all valid trials: 10 trials in 109 participants and nine in one participant after exclusion of a confirmed recording-process error.

For minimum-total-time scoring, decision and movement times were taken from the same trial as the minimum total time, not from their independently fastest trials. Levene p values are based on the mean. Kruskal–Wallis tests have 3 degrees of freedom and use asymptotic p values. η² and its 95% CI are from the conventional one-way ANOVA, not the Welch test. All p values are unadjusted.

ANOVA, analysis of variance; SD, standard deviation; CI, confidence interval; df, degrees of freedom. Primary minimum-total-time ANOVA results are presented in main Table 1.

Supplementary Table S2 Multiple linear regression models using mean trial values

Mean total, decision and movement times were analyzed in 110 participants.

| **Outcome** | **Predictor** | **B (SE)** | **β** | **95% CI for B** | **p** |
| --- | --- | --- | --- | --- | --- |
| Total time | Intercept | -0.094 (1.314) | — | -2.700 to 2.512 | 0.943 |
|  | Age, years | 0.042 (0.015) | 0.244 | 0.013 to 0.071 | 0.005 |
|  | 5STST time, s | 0.104 (0.020) | 0.418 | 0.064 to 0.144 | <0.001 |
|  | Handgrip strength, kg | -0.021 (0.010) | -0.241 | -0.041 to -0.001 | 0.041 |
|  | SMI, kg/m² | 0.061 (0.056) | 0.088 | -0.050 to 0.173 | 0.276 |
|  | FES-I score | 0.001 (0.005) | 0.008 | -0.010 to 0.011 | 0.918 |
|  | Sex, female vs male | -0.129 (0.158) | -0.097 | -0.441 to 0.184 | 0.416 |
| Decision time | Intercept | 0.138 (0.402) | — | -0.660 to 0.936 | 0.732 |
|  | Age, years | 0.013 (0.005) | 0.257 | 0.004 to 0.022 | 0.005 |
|  | 5STST time, s | 0.027 (0.006) | 0.372 | 0.015 to 0.040 | <0.001 |
|  | Handgrip strength, kg | -0.005 (0.003) | -0.206 | -0.011 to 0.001 | 0.089 |
|  | SMI, kg/m² | -0.004 (0.017) | -0.022 | -0.039 to 0.030 | 0.796 |
|  | FES-I score | -0.003 (0.002) | -0.164 | -0.006 to 0.000 | 0.051 |
|  | Sex, female vs male | 0.017 (0.048) | 0.044 | -0.078 to 0.113 | 0.719 |
| Movement time | Intercept | -0.232 (1.088) | — | -2.390 to 1.925 | 0.831 |
|  | Age, years | 0.029 (0.012) | 0.209 | 0.005 to 0.053 | 0.020 |
|  | 5STST time, s | 0.077 (0.017) | 0.383 | 0.043 to 0.110 | <0.001 |
|  | Handgrip strength, kg | -0.016 (0.008) | -0.224 | -0.032 to 0.001 | 0.064 |
|  | SMI, kg/m² | 0.066 (0.047) | 0.118 | -0.026 to 0.158 | 0.159 |
|  | FES-I score | 0.004 (0.004) | 0.071 | -0.005 to 0.013 | 0.395 |
|  | Sex, female vs male | -0.146 (0.130) | -0.137 | -0.405 to 0.113 | 0.265 |

Model fit for total, decision and movement time, respectively: R² = 0.356, 0.310 and 0.315; adjusted R² = 0.318, 0.269 and 0.275; F(6, 103) = 9.470, 7.696 and 7.893 (all model p < 0.001).

Means were calculated across all valid trials. Confidence intervals are conventional regression 95% CIs. Values are reproduced at the precision of the SPSS output; a displayed bound of 0.000 may reflect rounding.

All six covariates were entered simultaneously. Sex was coded 1 for male and 2 for female; its coefficient represents the adjusted difference for women relative to men. All p values in this table are unadjusted and are reported for sensitivity assessment; the primary FDR-adjusted results are presented in main Table 2.

5STST, five-times sit-to-stand test; SMI, skeletal muscle index measured by bioelectrical impedance analysis; FES-I, Falls Efficacy Scale–International; B, unstandardized coefficient; SE, standard error; β, standardized coefficient; CI, confidence interval; FDR, false discovery rate.

Supplementary Table S3 Bootstrap estimates for the primary regression models

Bootstrap inference was based on 5,000 simple resamples of the 110-participant analytic sample.

| **Outcome** | **Predictor** | **B** | **Bootstrap SE** | **BCa 95% CI** | **Bootstrap p** |
| --- | --- | --- | --- | --- | --- |
| Total time | Intercept | 0.438 | 1.214 | -1.918 to 2.798 | 0.718 |
|  | Age, years | 0.031 | 0.013 | 0.006 to 0.057 | 0.017 |
|  | 5STST time, s | 0.106 | 0.017 | 0.072 to 0.137 | <0.001 |
|  | Handgrip strength, kg | -0.016 | 0.008 | -0.032 to -0.002 | 0.051 |
|  | SMI, kg/m² | 0.042 | 0.046 | -0.047 to 0.122 | 0.367 |
|  | FES-I score | -0.002 | 0.005 | -0.011 to 0.010 | 0.677 |
|  | Sex, female vs male | -0.082 | 0.154 | -0.417 to 0.209 | 0.574 |
| Decision time | Intercept | 0.146 | 0.470 | -0.859 to 1.126 | 0.761 |
|  | Age, years | 0.009 | 0.005 | -0.001 to 0.019 | 0.105 |
|  | 5STST time, s | 0.026 | 0.006 | 0.013 to 0.034 | <0.001 |
|  | Handgrip strength, kg | -0.005 | 0.003 | -0.011 to 0.001 | 0.096 |
|  | SMI, kg/m² | 0.006 | 0.017 | -0.029 to 0.041 | 0.726 |
|  | FES-I score | 0.001 | 0.003 | -0.005 to 0.006 | 0.831 |
|  | Sex, female vs male | 0.018 | 0.043 | -0.073 to 0.101 | 0.688 |
| Movement time | Intercept | 0.292 | 1.110 | -1.738 to 2.378 | 0.793 |
|  | Age, years | 0.022 | 0.012 | -0.003 to 0.047 | 0.074 |
|  | 5STST time, s | 0.080 | 0.014 | 0.054 to 0.108 | <0.001 |
|  | Handgrip strength, kg | -0.011 | 0.007 | -0.024 to 0.002 | 0.118 |
|  | SMI, kg/m² | 0.036 | 0.041 | -0.043 to 0.106 | 0.371 |
|  | FES-I score | -0.003 | 0.006 | -0.014 to 0.011 | 0.659 |
|  | Sex, female vs male | -0.100 | 0.135 | -0.380 to 0.153 | 0.469 |

For minimum-total-time scoring, decision and movement times were taken from the same trial as the minimum total time, not from their independently fastest trials.

B is the coefficient in the original sample. BCa denotes bias-corrected and accelerated. Bootstrap p values and BCa intervals are distinct inferential summaries and are reproduced as reported by SPSS; they need not yield identical threshold decisions. For handgrip strength in the total-time model, the BCa interval excludes zero whereas the bootstrap p value is 0.051.

All six covariates were entered simultaneously. Sex was coded 1 for male and 2 for female; its coefficient represents the adjusted difference for women relative to men. All p values in this table are unadjusted and are reported for sensitivity assessment; the primary FDR-adjusted results are presented in main Table 2.

5STST, five-times sit-to-stand test; SMI, skeletal muscle index measured by bioelectrical impedance analysis; FES-I, Falls Efficacy Scale–International; B, unstandardized coefficient; SE, standard error; β, standardized coefficient; CI, confidence interval; FDR, false discovery rate.

Supplementary Table S4 Primary regression models after exclusion of one participant

The participant with the largest Cook’s distance in all three primary models was excluded, leaving 109 participants.

| **Outcome** | **Predictor** | **B (SE)** | **β** | **95% CI for B** | **p** |
| --- | --- | --- | --- | --- | --- |
| Total time | Intercept | 0.217 (1.142) | — | -2.048 to 2.482 | 0.850 |
|  | Age, years | 0.034 (0.013) | 0.220 | 0.008 to 0.060 | 0.010 |
|  | 5STST time, s | 0.105 (0.017) | 0.481 | 0.071 to 0.140 | <0.001 |
|  | Handgrip strength, kg | -0.017 (0.009) | -0.218 | -0.034 to 0.001 | 0.057 |
|  | SMI, kg/m² | 0.035 (0.049) | 0.057 | -0.062 to 0.131 | 0.474 |
|  | FES-I score | 0.001 (0.005) | 0.011 | -0.009 to 0.011 | 0.893 |
|  | Sex, female vs male | -0.083 (0.136) | -0.071 | -0.352 to 0.186 | 0.541 |
| Decision time | Intercept | 0.398 (0.355) | — | -0.306 to 1.103 | 0.265 |
|  | Age, years | 0.005 (0.004) | 0.123 | -0.002 to 0.013 | 0.176 |
|  | 5STST time, s | 0.026 (0.005) | 0.415 | 0.015 to 0.037 | <0.001 |
|  | Handgrip strength, kg | -0.004 (0.003) | -0.185 | -0.010 to 0.001 | 0.133 |
|  | SMI, kg/m² | 0.014 (0.015) | 0.081 | -0.016 to 0.044 | 0.345 |
|  | FES-I score | -0.002 (0.002) | -0.131 | -0.005 to 0.001 | 0.124 |
|  | Sex, female vs male | 0.020 (0.042) | 0.058 | -0.064 to 0.103 | 0.643 |
| Movement time | Intercept | -0.181 (0.971) | — | -2.107 to 1.745 | 0.852 |
|  | Age, years | 0.029 (0.011) | 0.225 | 0.007 to 0.050 | 0.011 |
|  | 5STST time, s | 0.079 (0.015) | 0.440 | 0.050 to 0.108 | <0.001 |
|  | Handgrip strength, kg | -0.013 (0.007) | -0.201 | -0.027 to 0.002 | 0.090 |
|  | SMI, kg/m² | 0.021 (0.041) | 0.041 | -0.061 to 0.103 | 0.620 |
|  | FES-I score | 0.003 (0.004) | 0.059 | -0.005 to 0.011 | 0.469 |
|  | Sex, female vs male | -0.103 (0.115) | -0.107 | -0.332 to 0.126 | 0.375 |

Model fit for total, decision and movement time, respectively: R² = 0.385, 0.287 and 0.341; adjusted R² = 0.348, 0.245 and 0.302; F(6, 102) = 10.627, 6.838 and 8.781 (all model p < 0.001).

For minimum-total-time scoring, decision and movement times were taken from the same trial as the minimum total time, not from their independently fastest trials.

In the primary models (N = 110), VIF values ranged from 1.040 to 2.240 and the minimum tolerance was 0.446. Durbin–Watson statistics were 1.739 for total time, 2.116 for decision time and 2.067 for movement time. Residual plots showed no substantial departures from linearity or homoscedasticity, although departures from normality were present in the distribution tails.

Maximum Cook’s distances in the primary models were 0.104 for total time, 0.827 for decision time and 0.438 for movement time. The same participant (study participant No. 27) had the largest Cook’s distance in all three models. This observation was retained in the primary analyses and excluded only in this sensitivity analysis; a large Cook’s distance does not by itself indicate a data-recording error.

The participant-level sensitivity exclusion is separate from removal of the confirmed erroneous final trial from another participant. Confidence intervals are conventional regression 95% CIs.

All six covariates were entered simultaneously. Sex was coded 1 for male and 2 for female; its coefficient represents the adjusted difference for women relative to men. All p values in this table are unadjusted and are reported for sensitivity assessment; the primary FDR-adjusted results are presented in main Table 2.

5STST, five-times sit-to-stand test; SMI, skeletal muscle index measured by bioelectrical impedance analysis; FES-I, Falls Efficacy Scale–International; B, unstandardized coefficient; SE, standard error; β, standardized coefficient; CI, confidence interval; FDR, false discovery rate.
